# Supplementary material for: Mechanisms governing the pioneering and redistribution capabilities of the non-classical pioneer PU.1
Source: Nat Commun. 2020 Jan 21;11:402. doi: 10.1038/s41467-019-13960-2 (PMC6972792; doi:10.1038/s41467-019-13960-2)
Supplement: Supplementary file 7 — Source data [file 41467_2019_13960_MOESM7_ESM.zip › Source_Data/FigureS2/FigureS2B_MotifScanOutput/KmeansCluster11/homerResults/motif3.similar.html]

motif3

## Information for motif3

A
T
C
G
G
C
A
T
C
T
G
A
T
C
G
A
A
G
T
C
A
T
G
C
C
T
G
A
T
G
A
C
C
T
G
A
C
T
G
A
  
Reverse Opposite:  

G
A
C
T
G
A
C
T
A
C
T
G
G
A
C
T
T
A
C
G
A
C
T
G
A
G
C
T
G
A
C
T
C
G
T
A
T
A
G
C
  

|  |  |
| --- | --- |
| p-value: | 1e-140 |
| log p-value: | -3.239e+02 |
| Information Content per bp: | 1.603 |
| Number of Target Sequences with motif | 920.0 |
| Percentage of Target Sequences with motif | 28.32% |
| Number of Background Sequences with motif | 5094.5 |
| Percentage of Background Sequences with motif | 11.27% |
| Average Position of motif in Targets | 101.9 +/- 52.0bp |
| Average Position of motif in Background | 99.8 +/- 62.0bp |
| Strand Bias (log2 ratio + to - strand density) | 0.0 |
| Multiplicity (# of sites on avg that occur together) | 1.15 |
| Motif File: | file (matrix) reverse opposite |

### Similar de novo motifs found

|  |  |  |  |  |  |  |  |
| --- | --- | --- | --- | --- | --- | --- | --- |
| Rank | Match Score | Redundant Motif | P-value | log P-value | % of Targets | % of Background | Motif file |
| 1 | 0.983 | A T C G A C G T G A T C C G A T A T C G G A C T A C T G A C T G A G C T A G C T C T A G | 1e-140 | -323.023024 | 29.42% | 12.04% | motif file (matrix) |
| 2 | 0.983 | G C A T C G T A T C G A A T G C T A G C C T G A A G T C G C T A | 1e-134 | -309.720610 | 28.35% | 11.58% | motif file (matrix) |
| 3 | 0.936 | C A G T G T C A G C A T A C T G G A C T A C T G A C T G A C G T A G C T | 1e-125 | -289.294354 | 36.20% | 17.78% | motif file (matrix) |
| 4 | 0.941 | G C T A C G T A T C G A A G T C A G T C C T G A A G T C G T C A T C G A | 1e-114 | -263.070311 | 14.34% | 3.86% | motif file (matrix) |
| 5 | 0.963 | A C G T A C T G A C G T A C T G A C T G A G C T G A C T C G A T A C T G C A T G C A T G A C G T | 1e-104 | -240.460680 | 11.60% | 2.77% | motif file (matrix) |
| 6 | 0.969 | C G T A C G T A A G T C A G T C C T G A A G T C C T G A | 1e-103 | -237.366732 | 15.64% | 4.92% | motif file (matrix) |
| 7 | 0.926 | G A C T C G A T A C T G A C G T A C T G A C T G A G C T | 1e-99 | -229.328272 | 27.27% | 12.69% | motif file (matrix) |
| 8 | 0.970 | C T A G G C T A C T G A C G T A A G T C A G T C C G T A T G A C G T C A T C G A T G C A T C A G G C A T | 1e-96 | -222.687745 | 10.83% | 2.60% | motif file (matrix) |
| 9 | 0.919 | G C A T C G T A T C G A T G A C A G T C T C G A A G T C | 1e-85 | -197.716199 | 42.47% | 25.89% | motif file (matrix) |
| 10 | 0.929 | C A G T A C G T C A G T A C T G G A T C C G A T A C T G C G A T A C T G A C T G A G C T A G C T C A G T A G C T | 1e-83 | -192.348703 | 14.04% | 4.73% | motif file (matrix) |
| 11 | 0.938 | A T G C G C A T A T G C C G A T A T C G C G A T A C T G C A T G A C G T A C G T C G T A C A T G A C T G C A G T | 1e-78 | -180.791230 | 15.02% | 5.53% | motif file (matrix) |
| 12 | 0.837 | G C A T C T G A A G T C A T G C C T A G T G A C C T G A T C G A C A T G | 1e-62 | -144.311913 | 15.08% | 6.33% | motif file (matrix) |
| 13 | 0.886 | G T A C A G C T C G A T A C T G A G C T A C T G A C T G A G C T | 1e-54 | -124.481773 | 11.67% | 4.57% | motif file (matrix) |
| 14 | 0.822 | A C G T A C T G A G C T A C T G A C T G A T G C C G A T | 1e-33 | -76.992381 | 22.04% | 13.84% | motif file (matrix) |
| 15 | 0.763 | A G T C C G T A C T A G C G T A A G T C A G T C C T G A A G T C C T G A | 1e-31 | -72.446518 | 3.54% | 0.85% | motif file (matrix) |
| 16 | 0.779 | T C G A T G C A T C G A C G T A A G T C A G T C A T C G A T G C C T G A T G C A T A G C T A C G | 1e-15 | -36.325252 | 2.52% | 0.82% | motif file (matrix) |
